# The Effectiveness of Electromyographic Biofeedback in Reducing Musculoskeletal Pain: A Systematic Review and Meta-Analysis

**DOI:** 10.3390/healthcare14213723

**Published:** 2026-11-02

**Authors:** Sotiria Vrouva, Nikolaos Ksimitiris, Nefeli Denaxa, Konstantinos Chanopoulos, George A. Koumantakis, George Gioftsos

**Affiliations:** 1 Physiotherapy Department, School of Health and Care Sciences, University of West Attica (UNIWA), 12243 Athens, Greece; phys22683071@uniwa.gr (N.K.); phys22683130@uniwa.gr (N.D.); gkoumantakis@uniwa.gr (G.A.K.); gioftsos@uniwa.gr (G.G.); 2 Department of Application Developments, Hellenic Military Geographical Service, 11362 Athens, Greece; k_hanos@yahoo.com

**Keywords:** Biofeedback, efficacy, physiotherapy, systematic review, meta-analysis

## Abstract

Background: Despite the ongoing debate surrounding its analgesic efficacy, EMG-BF(Electromyographic Biofeedback) is commonly used for neuromuscular re-education. This study aimed to quantify its pain-reducing effects. Methods: Two researchers followed PRISMA 2020 guidelines to synthesize randomized controlled trials comparing EMG-BF to control interventions. By using a random-effects model, they calculated standardized mean differences (Hedges’ *g*). A total of 9 studies were included in the systematic review, and 8 of these were subjected to meta-analysis. The assessment of risk of bias and certainty of evidence was done using Cochrane RoB 2 and the GRADE approach, respectively. Results: Eight trial comparisons (*N* = 298 participants; 149 experimental, 149 control) evaluating continuous clinical pain intensity met all inclusion criteria. EMG biofeedback demonstrated a statistically significant reduction in pain intensity compared to control interventions (random-effects SMD = −0.6046, 95% CI: [−0.8707; −0.3384], *z* = −4.45, *p* < 0.0001; common-effect SMD = −0.6193, 95% CI: [−0.8541; −0.3844], *p* < 0.0001). Between-study heterogeneity was low and non-significant (*I*^2^ = 17.7%, *τ*^2^ = 0.0259, *Q* = 8.50, *df* = 7, *p =* 0.2905). The 95% prediction interval remained entirely below zero [−1.1026; −0.1065]. Subgroup analysis by pain tool (7 VAS comparisons, 1 NPRS comparison) showed no significant interaction (*Q* = 1.66, *p* = 0.1973). Egger’s linear regression test indicated no significant small-study effect (*t* = 1.17, *p* = 0.2874). Conclusion: These findings indicate that EMG-BF provides clinically meaningful pain reduction in musculoskeletal disorders, especially when used alongside conventional physical therapy.

## 1. Introduction

Electrophysical agents represent established components of physical rehabilitation, delivering thermal, mechanical, or electromagnetic energy to biological tissues [1,2,3]. This helps in reducing pain, supporting tissue healing, and restoring neuromuscular function [1,2,3]. While traditional passive methods are commonly used either independently or together with therapeutic exercises, biofeedback has emerged as an active approach for neuromuscular re-education over the past fifty years [4]. Unlike passive treatments, biofeedback enables individuals to regulate operant neuromuscular responses through continuous sensory feedback, enhancing motor control and promoting functional mobility [4,5,6,7,8].

Electromyographic biofeedback (EMG-BF) utilizes surface electrodes placed over target muscles to detect microvolt-level electrical activity without delivering external electrical stimulation [4,9,10,11]. These signals are processed through amplification, conversion to digital format, and filtering to eliminate artifacts, before being presented in real-time through computer screens or interactive sound interfaces as feedback [4,9,10,11].

Biofeedback techniques cover various physiological aspects like electromyographic, thermal, electrodermal, respiratory, heart rate variability, and neurofeedback, as well as biomechanical systems, targeting autonomic balance, neural adaptability, and motor unit control mechanisms [4,10,12].

In clinical settings, biofeedback disrupts the sustained pain-spasm vicious cycle by modulating motor unit firing rates in neurological, pelvic floor, and orthopedic rehabilitation fields [4,10,11]. In musculoskeletal therapy, it facilitates targeted motor retraining of specific muscle groups, enhancing spinal and scapular motor functions, and managing chronic pain conditions such as lower back pain, fibromyalgia, and temporomandibular disorders [4,10,11]. This becomes even more effective when combined with modern wearable technologies and interactive platforms [4,10,11,13].

According to the International Association for the Study of Pain (IASP), pain is defined as an unpleasant sensory and emotional experience associated with, or resembling that associated with, actual or potential tissue damage. Differentiating subacute pain (typically resolving within 3 months alongside tissue healing) from chronic pain is critical, as chronic presentations are predominantly driven by central sensitization, neuroplastic alterations, and cognitive affective mechanisms rather than pure structural pathology [14]. Musculoskeletal pain conditions represent a leading contributor to global disability burden, frequently characterized by persistent muscle tension, impaired muscle coordination, and irregular motor unit activation. EMG-BF directly addresses these issues by promoting active muscle relaxation and motor skills relearning [4,10,11,13,15]. Nonetheless, there is variability in ongoing clinical studies concerning comparison methods, treatment parameters, and research quality. Therefore, systematic analysis and synthesis were carried out to evaluate the effectiveness of EMG-BF in alleviating pain and improving pain tolerance in adult musculoskeletal patients compared to standard interventions.

## 2. Materials and Methods

### 2.1. Study Design and Protocol Registration

This review and meta-analysis followed the guidelines outlined in the Preferred Reporting Items for Systematic Reviews and Meta-Analyses (PRISMA 2020) statement and the methodological principles detailed in the Cochrane Handbook for Systematic Reviews of Interventions [16,17].

The inclusion criteria were defined using the PICO framework [17]:

- Population: Adults (≥18 years old) diagnosed with subacute or chronic musculoskeletal pain conditions.
- Intervention: Utilization of surface electromyographic biofeedback (EMG-BF) as a standalone treatment method or as part of structured active physical therapy and therapeutic exercise programs.
- Comparator: Control groups receiving traditional passive physical therapy, active therapeutic exercises, stationary cycling, or sham/placebo treatments.
- Outcomes: The main focus was on changes in pain intensity post-treatment, evaluated through the Visual Analog Scale (VAS) or Pressure Pain Threshold (PPT, measured in kg/cm^2^).

Study Design: Inclusion criteria encompassed peer-reviewed randomized controlled trials (RCTs) released from 2015 to 2026.

### 2.2. Evaluation of Methodological Rigor and Bias Risk

This systematic review and meta-analysis were conducted and reported in strict accordance with the Preferred Reporting Items for Systematic Reviews and Meta-Analyses (PRISMA 2020) statement. The study protocol was prospectively designed and registered with the Open Science Framework (OSF registration ID: https://doi.org/10.17605/OSF.IO/2KHYZ, accessed on 25 August 2026). Registration was initiated prior to formal title/abstract screening and completed before formal data extraction to prevent post-hoc analytical deviations.

A comprehensive systematic literature search was executed across three electronic bibliographic databases: PubMed/MEDLINE, ScienceDirect, and Google Scholar, covering records published from January 2015 up to August 2026 (final search update performed on 24 August 2026). In addition, backward and forward citation tracking (snowball search) of eligible trials and pertinent review articles was manually performed to ensure complete retrieval.

The search syntax combined Medical Subject Headings (MeSH) and free-text terms structured around three conceptual domains: (1) intervention modality, (2) target condition, and (3) pain outcomes. The overarching search string utilized across databases was

(“electromyographic biofeedback” OR “EMG biofeedback” OR “surface EMG biofeedback” OR “sEMG biofeedback” OR “electromyography feedback”) AND (“musculoskeletal pain” OR “chronic pain” OR “patellofemoral pain syndrome” OR “knee osteoarthritis” OR “chronic low back pain” OR “neck pain” OR “myofascial pain syndrome” OR “arthralgia”) AND (“pain intensity” OR “Visual Analogue Scale” OR “VAS” OR “Numeric Pain Rating Scale” OR “NPRS” OR “pressure pain threshold” OR “PPT” OR “randomized controlled trial”).

### 2.3. Search Restrictions and Screening Limits

Searches were restricted to peer-reviewed randomized controlled trials published in English. For the Google Scholar database, search output was organized by algorithmic relevance, and screening was truncated at the first 200 consecutive records. This pre-specified restriction was implemented because empirical evidence demonstrates that relevance-ranked screening beyond 200–300 records in gray-literature search engines yields negligible sensitivity gains while disproportionately increasing false-positive noise.

All retrieved citations (*Ν* = 1059) were imported into EndNote reference management software for systematic deduplication and screening. Following duplicate removal (*n* = 483), two investigators independently screened the titles and abstracts of 576 unique records, excluding 548 records that did not meet the PICO criteria.

The full texts of the remaining 28 potentially eligible articles were retrieved and scrutinized independently by both reviewers. Disagreements were resolved through consensus or adjudication by a third senior reviewer. Of these, 20 articles were excluded with study-specific rationale:

Ineligible comparator (e.g., biofeedback combined with active co-interventions without an isolated comparison group; *n* = 7). Non-randomized, quasi-experimental, or observational design (*n* = 5). Ineligible target population or non-musculoskeletal pathology (*n* = 4). Insufficient numeric data for change-score derivation with no response from study authors (*n* = 3). Re-analysis or secondary report of an already included trial cohort (*n* = 1).

### 2.4. Missing Data Retrieval Efforts

For studies with incomplete reporting of baseline-to-endpoint variance parameters, standard deviations of change scores, or exact participant retention figures, formal inquiries were addressed to the respective corresponding authors via email. Authors were given a standard three-week window to provide primary numeric datasets.

**Figure 1:**
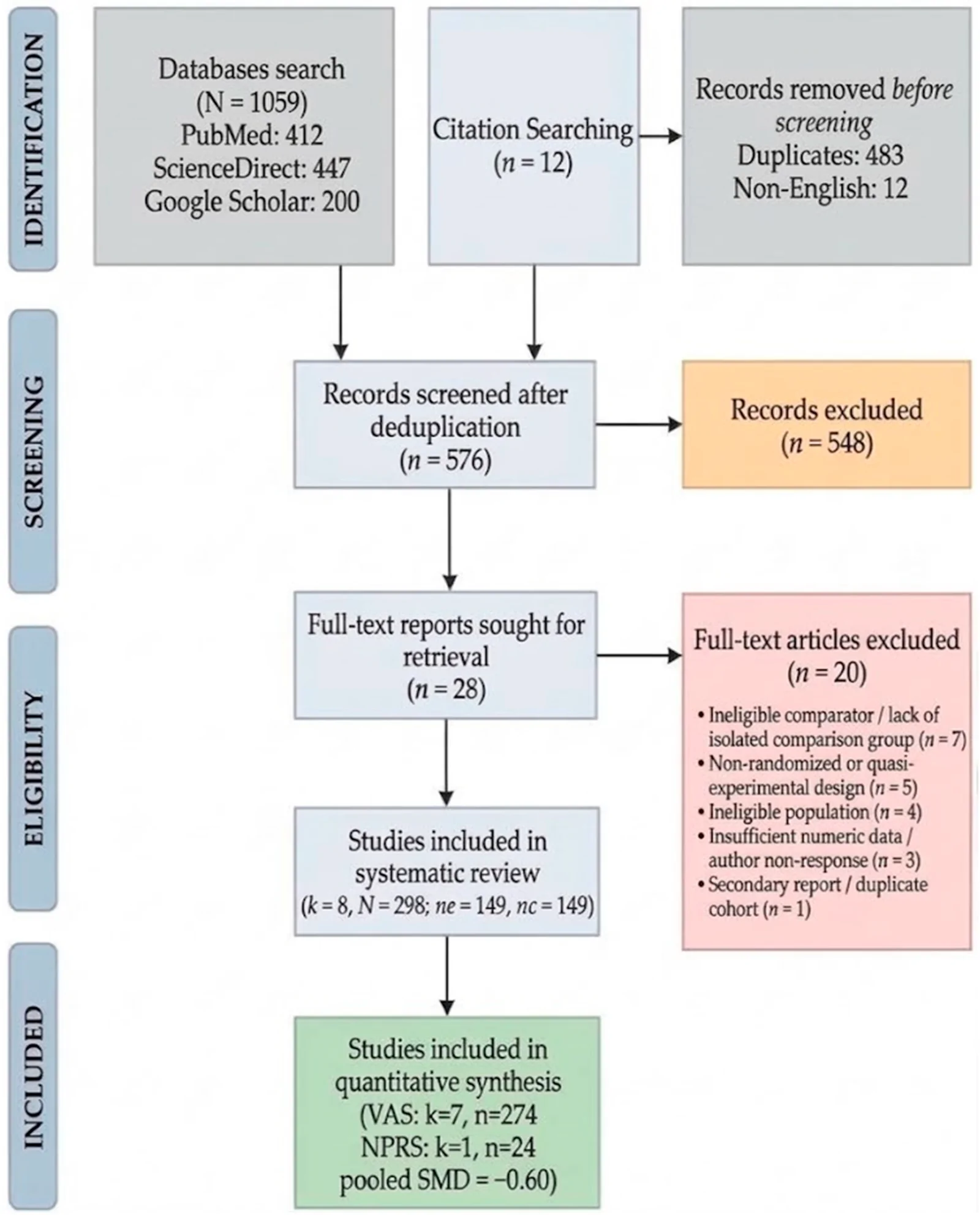
PRISMA 2020 flow diagram illustrating the identification, screening, full-text eligibility evaluation, and final inclusion of randomized controlled trials in the meta-analysis (*k* = 8, *N* = 298).

Ultimately, eight randomized controlled trials (*k* = 8, comprising 298 participants) fully satisfied all eligibility requirements and were incorporated into the final quantitative synthesis (Figure 1).

Methodological quality was appraised using the 10-item Physiotherapy Evidence Database (PEDro) scale as an auxiliary, secondary assessment of overall trial quality and reporting standards. We clarify that, contrary to earlier protocol phrasing, a PEDro score of ≥6/10 was not employed as an absolute, deterministic exclusion threshold for quantitative synthesis; rather, all eligible randomized trials meeting the predefined PICO eligibility criteria were preserved to prevent selection bias and maintain comprehensive meta-analytic coverage. Formal domain-level risk-of-bias evaluation for the synthesis and GRADE certainty profiling was conducted strictly using the Cochrane Risk of Bias 2 (RoB 2) tool (Table 1).

#### 2.4.1. Eligibility Criteria and Study Selection

Inclusion criteria were defined as:

A. Parallel-group randomized controlled trials involving adults over 18 years of age diagnosed with subacute or chronic musculoskeletal pain (symptom duration ≥1 month).
B. An intervention arm utilizing surface EMG biofeedback.
C. A concurrent control arm receiving conventional physical therapy, exercise, or sham/placebo.
D. Quantitative assessment of pain intensity at baseline and post-treatment using validated instruments, such as the Visual Analog Scale (VAS 0–10 cm) or Pressure Pain Threshold/dolorimeter (PPT, kg/cm^2^).

Exclusion criteria encompassed non-randomized studies, observational designs, trials lacking sufficient numerical data for effect size calculation (means, standard deviations, sample sizes), and studies evaluating biofeedback modalities other than electromyography (e.g., electroencephalographic neurofeedback, thermal biofeedback).

#### 2.4.2. Data Extraction and Systematic Review Synthesis

Data extraction was performed using a standardized coding form. Extracted variables comprised author names, publication year, sample size per treatment arm, anatomical condition treated, specific protocols for intervention and control groups, intervention duration, frequency of sessions, follow-up timelines, outcome metrics, and raw baseline to post-treatment pain scores. The qualitative synthesis systematically mapped the clinical heterogeneity of included trials, comparing intervention parameters, comparative control strategies, and within-group versus between-group statistical findings across studies.

#### 2.4.3. Risk of Bias and Certainty of Evidence Assessment

The methodological quality of included RCTs was evaluated using the revised Cochrane Risk of Bias tool for randomized trials (RoB 2) [27]. The appraisal covered five core domains: bias arising from the randomization process (D1), bias due to deviations from intended interventions (D2), bias due to missing outcome data (D3), bias in measurement of the outcome (D4), and bias in selection of the reported result (D5). Each domain and overall trial quality were graded as “Low risk”, “Some concerns”, or “High risk”. The overall certainty of evidence was appraised using the Grading of Assessment, Development and Evaluation (GRADE) methodology across the domains of risk of bias, inconsistency, indirectness, imprecision, and publication bias [28].

#### 2.4.4. Meta-Analytic Statistical Methods

Effect sizes for continuous pain outcomes were quantified as Standardized Mean Differences (SMD) expressed as Hedges’ *g* alongside corresponding 95% Confidence Intervals (95% CI) to correct for small-sample bias [29]. Across all trials, negative effect estimates denote superior pain reduction favouring the EMG-BFB intervention compared to control conditions. Statistical synthesis was conducted using the DerSimonian–Laird random-effects model to incorporate both within-study sampling error and between-study variance *(τ*^2^*)* [30]. A common-effect (fixed-effect) inverse-variance model was simultaneously computed for comparative weighting verification. Statistical heterogeneity was evaluated using the *I*^2^ statistic, between-study variance (*τ*^2^), and Cochran’s *Q* test (with significance set at *p* < 0.10). In addition, a 95% Prediction Interval (95% PI) was derived using Student’s *t*-distribution (*df* = 7) [17] to reflect the expected dispersion of effect sizes in future comparable clinical settings. Publication bias and small-study effects were evaluated via visual inspection of the funnel plot, Egger’s linear regression test [31], and Duval and Tweedie’s Trim-and-Fill method (using the *L*-estimator) [32], as well as Rosenthal’s [33] and Orwin’s [34] fail-safe N metrics. Sensitivity analyses comprised iterative Leave-One-Out models to evaluate individual study influence on pooled effect estimates and heterogeneity. Predefined subgroup analyses were stratified by pain measurement instrument [Visual Analogue Scale (VAS) vs. Numeric Pain Rating Scale (NPRS)]. Interpretation of clinical significance was primarily benchmarked [35] against between-group effect sizes and their associated uncertainty, where an SMD of approximately 0.50 represents a moderate between-group effect. Within-group mean reductions (e.g., changes from baseline) were treated strictly as descriptive context and were not used to infer comparative clinical superiority. Given that pooled effect estimates reflect standardized between-group differences, claims regarding treatment superiority were qualified by the width of the confidence and prediction intervals rather than assumed mechanistically.

## 3. Results

### 3.1. Systematic Review: Study Characteristics and Qualitative Synthesis

The systematic review and updated quantitative synthesis identified nine randomized controlled trials of which eight met all criteria for quantitative synthesis, published between 2015 and 2025, enrolling a cumulative total of 298 patients (149 in experimental EMG-BF groups and 149 in control groups) [18,19,21,22,23,24,25,26]. Trial sample sizes ranged from 14 to 90 participants. Anatomical regions evaluated across the included trials comprised the lumbar spine, knee joint, cervical region, and shoulder complex. Intervention durations ranged from 4 to 8 weeks. Seven trials assessed pain intensity using the Visual Analog Scale (VAS, 0–10 cm) and one trial utilized the Numeric Pain Rating Scale (NPRS, 0–10). Regarding construct divergence and directional coding discrepancies, trials evaluating mechanical pain sensitivity thresholds (i.e., Pressure Pain Threshold, PPT) were excluded from the pooled pain intensity synthesis and reserved for separate descriptive evaluation.

In the qualitative evaluation of individual trials: Trials evaluating EMG-BF alongside or against conventional/multimodal physical therapy protocols (Ahmed et al., 2016 [19]; Choi et al., 2015 [18]; Khojasteh et al., 2024 [24]; Porwal et al., 2023 [23]; Raeissadat et al., 2018 [21]; Sabir et al., 2026 [26]) reported substantial descriptive within-group pain relief across both treatment arms. Specifically, mean within-group reductions reached −5.13 ± 0.86 in Ahmed et al. (vs. −4.51 ± 0.70 in controls) [19], −3.79 ± 0.81 in Porwal et al. (vs. −2.89± 0.74) [23], −3.00 ± 1.65 in Raeissadat et al. (vs. −1.80 ± 1.44) [21], −4.48 ± 1.46 in Sabir et al. (vs. −4.08 ± 0.80) [26], −3.65 ± 2.65 in Khojasteh et al. (vs. −2.12 ± 2.41) [24], and −1.90± 1.83 in Choi et al. (vs. −0.40 ± 1.51) [18].

In trials where comparator arms received structured active exercise regimens, notable within-group improvements were observed in both arms (descriptive within-group changes of −4.60 ± 1.57 vs. −4.40 ± 2.05 in Oliveira et al., 2022 [22]; and −0.49 ± 2.63 vs. −1.40 ± 2.63 in Chang et al., 2025 [25]). The detailed trial characteristics, baseline parameters, and complete post-intervention data extraction are summarized in Table 2 and fully detailed in Supplementary File.

### 3.2. Methodological Quality Assessment (RoB 2)

**Figure 2:**
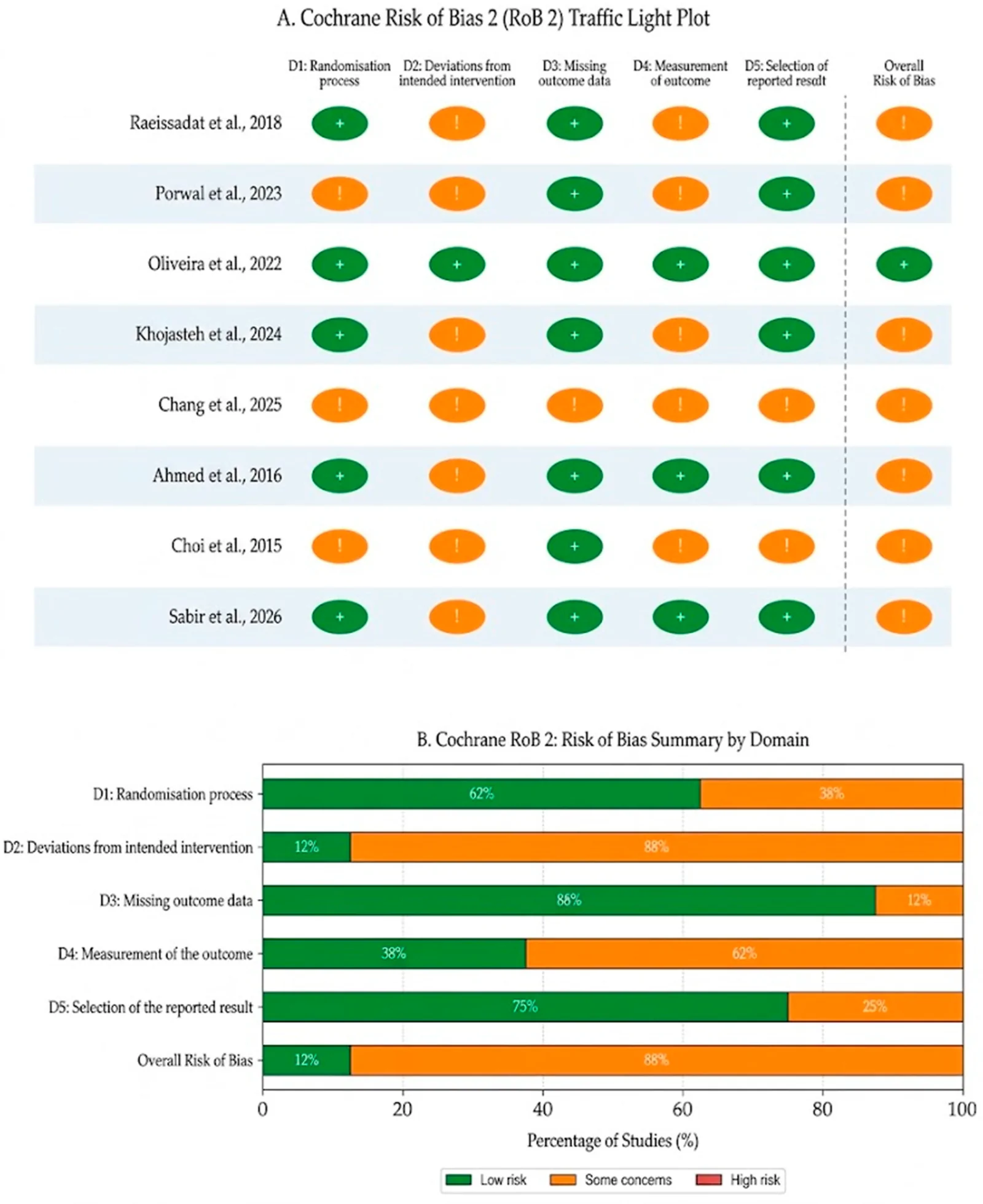
Methodological quality and risk-of-bias appraisal using the Cochrane RoB 2 tool across included randomized controlled trials (*k* = 8) [18,19,21,22,23,24,25,26]: (**A**) Traffic-light plot depicting outcome-specific domain-level judgments for each individual trial; (**B**) summary weighted bar plot illustrating the proportional distribution of risk of bias across each domain and overall. Notes: Green circles with plus signs (+) denote low risk of bias; orange circles with exclamation marks (!) denote some concerns; red circles with minus signs (−) denote high risk of bias. D1: Randomisation process; D2: Deviations from intended interventions; D3: Missing outcome data; D4: Measurement of the outcome; D5: Selection of the reported result. Domain 3 reflects trial-specific attrition (e.g., 33.3% loss to follow-up in Chang et al., 2025 [25]); Domain 4 reflects participant awareness during subjective pain scoring (VAS/NPRS).

Methodological quality appraisal utilizing the Cochrane Risk of Bias 2 (RoB 2) tool across the eight included randomized controlled trials revealed that seven trials (87.5%) were judged as having “Some concerns” overall, while one trial (12.5%) was classified as having “Low risk” of bias (Figure 2). Across individual domains, methodological strengths and limitations were distributed as follows:

Domain 1 (Randomization process): Five trials (62.5%) demonstrated low risk of bias, reporting computer-generated random sequence generation and centralized or sealed envelope allocation concealment. Three trials (37.5%) were judged as having some concerns due to insufficient details regarding the specific concealment mechanisms utilized prior to assignment.

Domain 2 (Deviations from intended interventions): Seven trials (87.5%) raised some concerns, reflecting the inherent clinical challenge of blinding participants and treating physiotherapists during active neuromuscular biofeedback or exercise interventions (participant awareness of assigned arm). One trial (12.5%) was rated as low risk due to balanced protocol delivery, verified intervention adherence, and standard co-intervention monitoring.

Domain 3 (Missing outcome data): Seven trials (87.5%) exhibited low risk of bias, maintaining complete follow-up or negligible, balanced attrition. In contrast, one trial (Chang et al., 2025 [25]) was judged as having some concerns regarding missing outcome data, as only 14 of the 21 randomized participants completed endpoint evaluations (representing a 33.3% overall attrition rate). Consequently, previous assertions of uniformly negligible attrition across all included trials were corrected, and this outcome attrition was incorporated into sensitivity considerations.

Domain 4 (Measurement of the outcome): Five trials (62.5%) were judged as having some concerns, arising from the outcome-specific assessment of pain intensity via subjective patient-reported scales (VAS or NPRS) by unblinded participants who were aware of their treatment allocation. The remaining three trials (37.5%) demonstrated low risk, having incorporated blinded outcome assessors for all post-intervention clinical assessments.

Domain 5 (Selection of the reported result): Six trials (75.0%) were deemed low risk, demonstrating concordance with pre-specified endpoints, whereas two trials (25.0%) were rated as having some concerns due to the absence of publicly available prospective trial registrations or published statistical analysis plans, precluding definitive exclusion of selective outcome reporting.

### 3.3. Meta-Analysis of Primary Outcomes and Prediction Interval

Across the eight included randomized controlled trials comprising a cumulative total of 298 participants (*n_e_* = 149 in experimental EMG-BF cohorts and *n_c_* = 149 in control cohorts), the meta-analytic synthesis demonstrated a statistically significant advantage for EMG-BF in reducing musculoskeletal pain intensity compared to control interventions. Under the common-effect inverse-variance model, the pooled effect yielded a statistically significant pain reduction (SMD = −0.6193, 95%CI: [−0.8541, −0.3844], *z* = −5.17, *p* < 0.0001). When evaluating the data under the DerSimonian–Laird random-effects model to account for between-study variance, the overall treatment effect remained robust and statistically significant (SMD = −0.6046, 95%CI: [−0.8707, −0.3384], *z* = −4.45, *p* < 0.0001).

Individual study-level estimates revealed substantial and statistically significant pain reductions favoring EMG-BF in trials utilizing structured physical therapy comparators, specifically Ahmed et al. (2016) (SMD = −0.7840, 95% CI: [−1.2134, −0.3545] [19], random weight: 25.0%), Raeissadat et al. (2018) (SMD = −0.7586, 95% CI: [−1.3947, −0.1224] [21], random weight: 14.1%), and Porwal et al. (2023) (SMD = −1.1351, 95%CI: [−1.8552, −0.4150], random weight: 11.5%) [23]. Smaller cohorts also exhibited point estimates favoring the experimental intervention, though their corresponding 95% confidence intervals spanned the line of no effect due to limited sample sizes: Choi et al. (2015) (SMD = −0.8562, 95%CI: [−1.7808, 0.0684] [18], random weight: 7.4%), Khojasteh et al. (2024) (SMD = −0.5877, 95% CI: [−1.3208, 0.1454] [24], random weight: 11.1%), and Sabir et al. (2016) (SMD = −0.3337, 95% CI: [−0.9291, 0.2618], random weight: 15.6%) [26]. Conversely, in trials where control groups received structured active exercise regimens, between-group standardized differences were non-significant, with confidence intervals spanning zero in both Oliveira et al. (2022) (SMD = −0.1058, 95%CI: [0.9066, 0.6951], random weight: 9.6%) and Chang et al. (2025) (SMD = 0.3238, 95%CI: [0.7331, 1.3808], random weight: 5.8%) [22,25].

**Figure 3:**
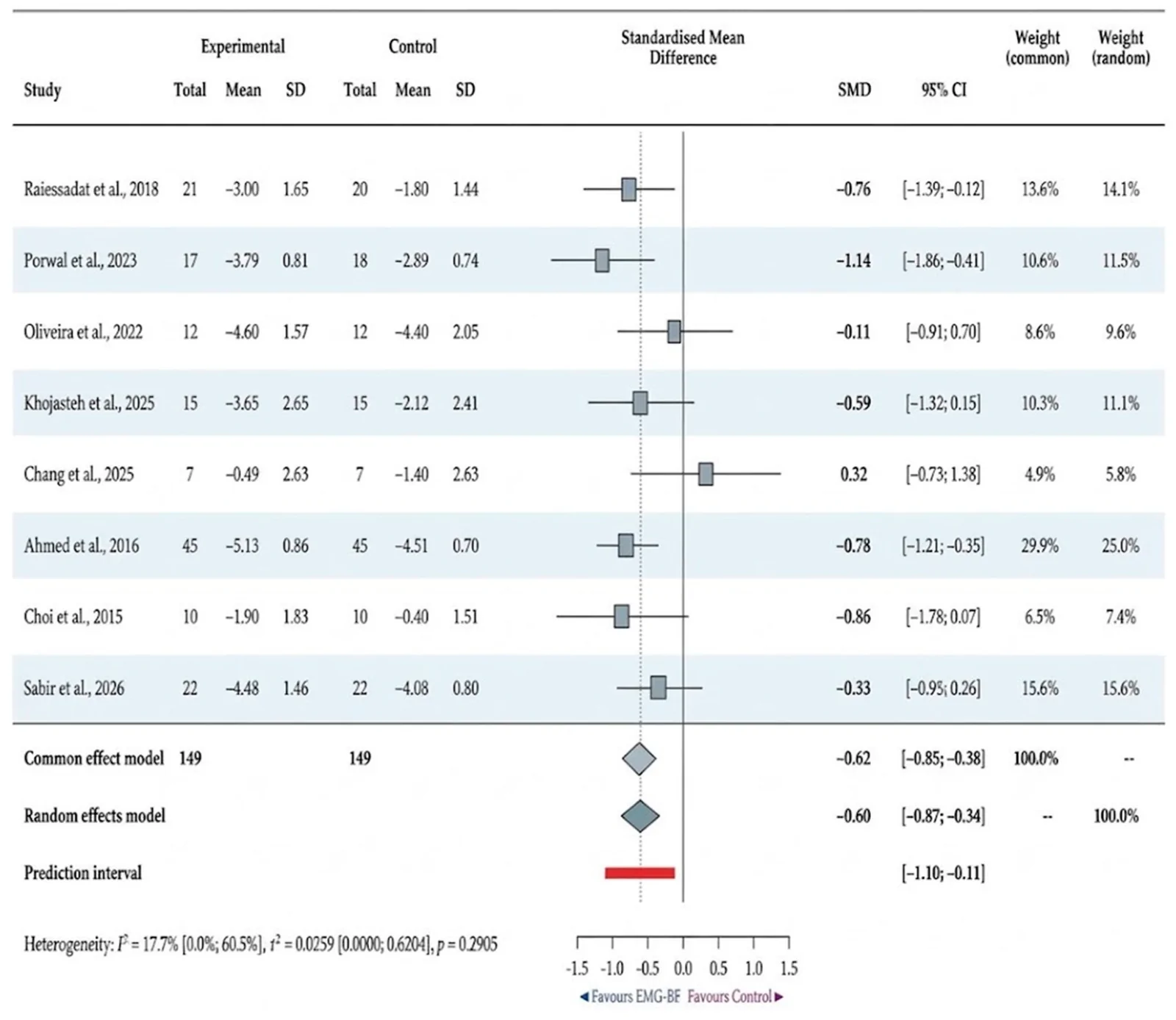
Forest plot of standardized mean differences (Hedges’ *g)* and 95% confidence intervals comparing EMG Biofeedback versus control interventions for pain intensity (*k* = 8, *N* = 298) [18,19,21,22,23,24,25,26]. The vertical dashed line represents the overall pooled effect estimate of the meta-analysis (SMD = −0.60).

Assessment of statistical heterogeneity across all eight trials demonstrated low-to-moderate, non-significant between-study variance (*τ*^2^= 0.0259, 95%CI: [0.0000, 0.6204]; *τ* = 0.1610, 95% CI: [0.0000, 0.7876]; *I*^2^ = 17.7%, 95% CI: [0.0%, 60.5%]; Cochran’s *Q* = 8.50, *df* = 7, *p* = 0.2905). The derived 95% prediction interval (based on the t-distribution, *df* = 7) ranged from −1.1026 to −0.1065. Because this prediction interval remains entirely below zero, it indicates that a beneficial pain reduction favoring EMG-BF is expected to be maintained across future comparable clinical implementations (Figure 3).

### 3.4. Statistical Weight Distribution and Clinical Meaningfulness (MCID)

**Figure 4:**
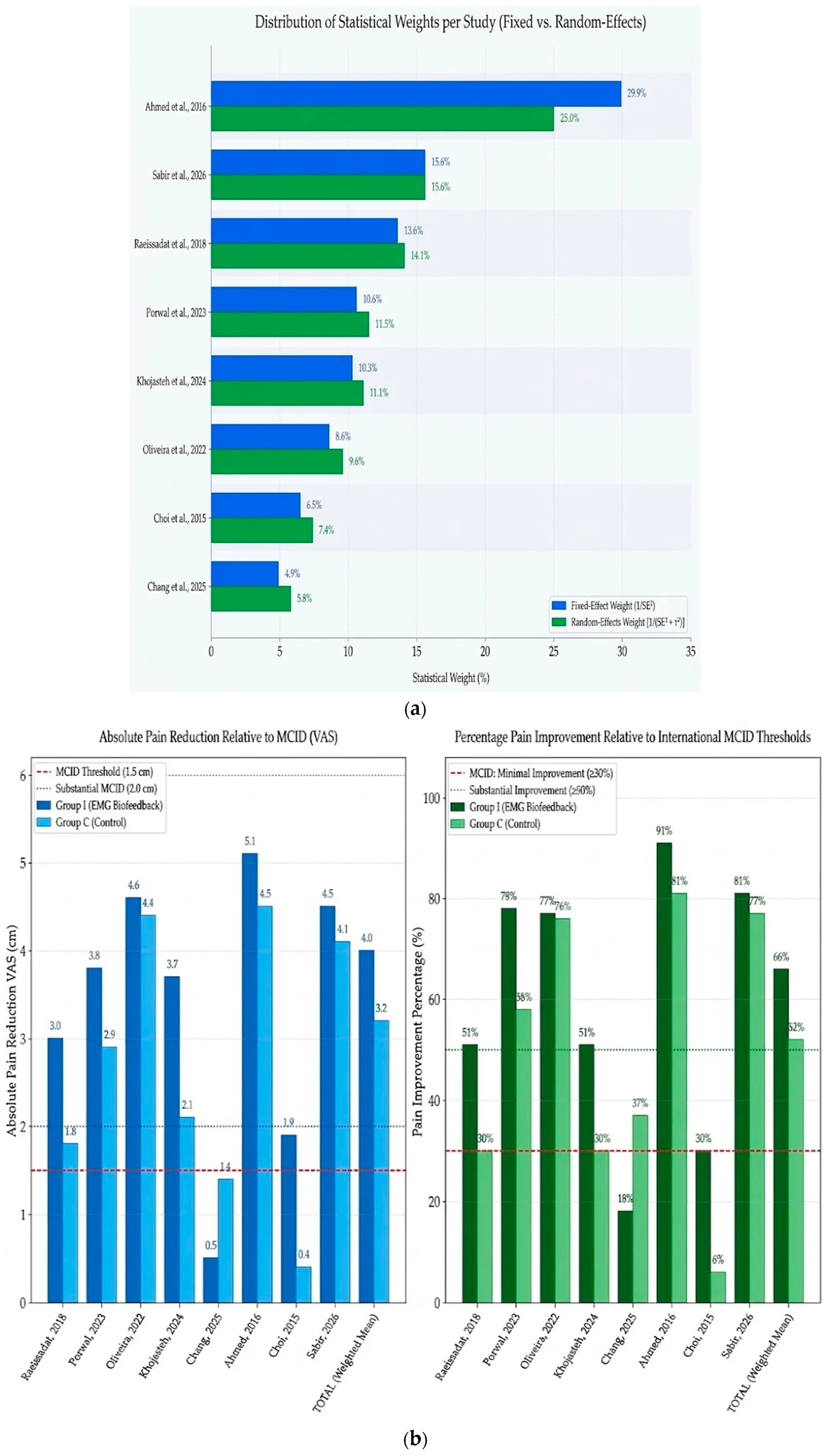
(**a**) Distribution of statistical weights per study under Fixed-Effect () and Random-Effects () models across the included trials (*k* = 8) [18,19,21,22,23,24,25,26]. (**b**) Clinical meaningfulness assessment of EMG Biofeedback compared to control interventions across included trials.

Statistical Weight Distribution and Clinical Meaningfulness in the quantitative synthesis (*k* = 8, *N* = 298): Statistical weight was distributed across trials with varying sample sizes and variances. Under the DerSimonian–Laird random-effects model, the largest analytical weights were assigned to Ahmed et al. (2016) [19] (25.0%), Sabir et al. (2026) [26] (15.6%), and Raeissadat et al. (2018) [21] (14.1%), followed by Porwal et al. (2023) [23] (11.5%), Khojasteh et al. (2024) [24] (11.1%), Oliveira et al. (2022) [22] (9.6%), Choi et al. (2015) [18] (7.4%), and Chang et al. (2025) [25] (5.8%). Given the low between-study variance (*τ*^2^ = 0.0259, *I*^2^ = 17.7%, *p* = 0.2905), weight distribution remained largely comparable between the common-effect and random-effects frameworks, precluding disproportionate distortion by smaller cohorts. Clinical meaningfulness was interpreted primarily through standardized between-group effect sizes, and their associated uncertainty bounds rather than within-group baseline changes. The pooled random-effects estimate of SMD = −0.6046 (95%CI: [−0.8707, −0.3384], *p* < 0.0001) surpassed the conventional benchmark for a moderate between-group effect (SMD ≈ 0.50). Furthermore, the derived 95% prediction interval ranged from −1.1026 to −0.1065, remaining entirely below zero and confirming that the favorable pain-relieving effect of EMG-BFB is expected to persist in future clinical settings. Although raw within-group pain reductions in several intervention arms descriptively exceeded established Minimal Clinically Important Difference (MCID) thresholds (e.g., reductions ≥ 1.5 cm on the VAS), parallel improvements within control cohorts necessitated that comparative clinical superiority be inferred strictly from between-group contrasts. Exploratory evaluation across comparator types confirmed that while EMG-BFB provides a distinct clinical advantage over conventional passive physiotherapy, its comparative superiority over structured active exercise regimens remains inconclusive (Figure 4a,b).

### 3.5. Publication Bias and Small-Study Effects

**Figure 5:**
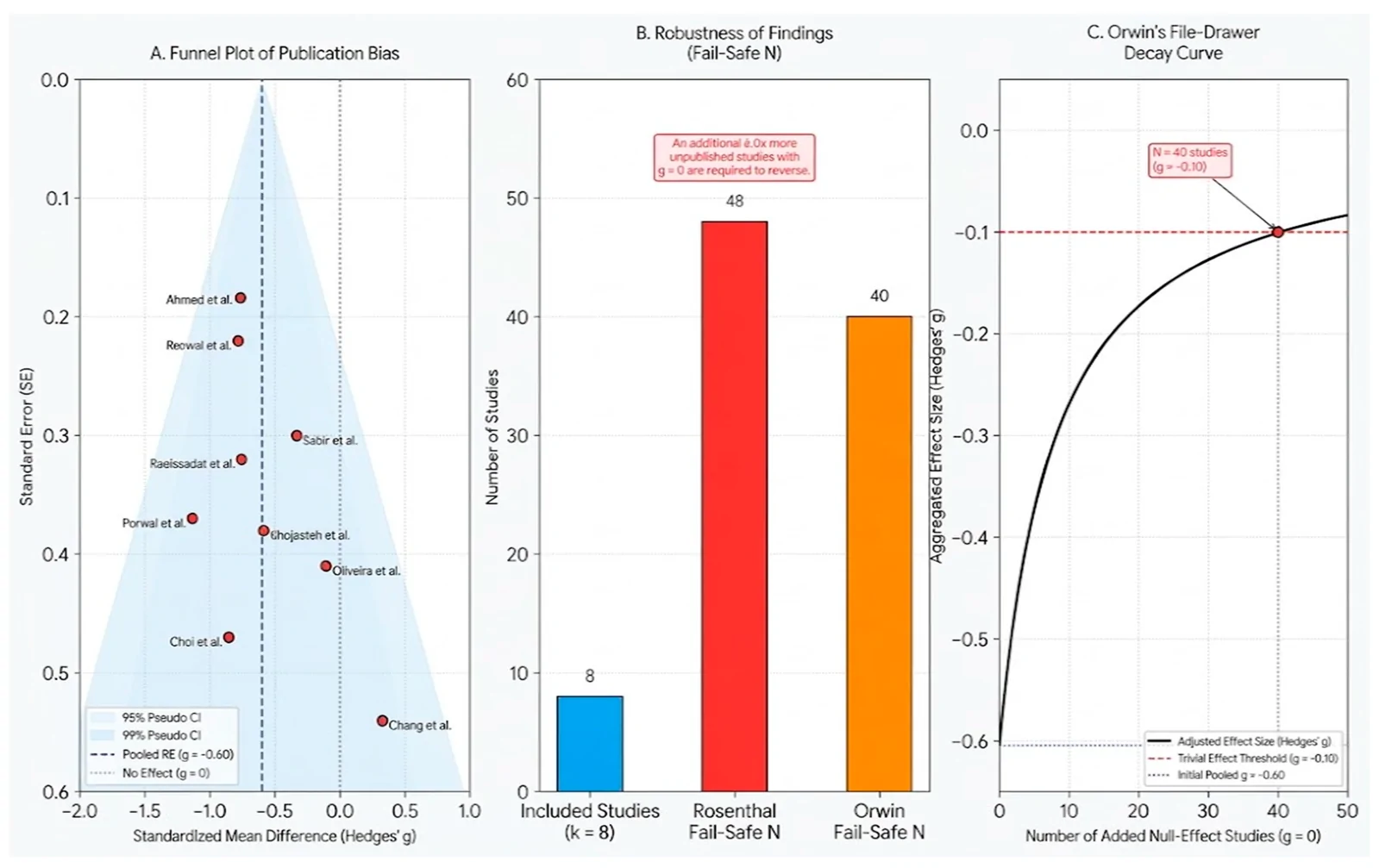
Publication bias and small-study effect diagnostics for pain intensity reduction (*k* = 8, *N* = 298) [18,19,21,22,23,24,25,26]. (**A**) Funnel plot of effect size (Hedges’ *g*) versus standard error demonstrating general distribution symmetry around the pooled random-effects estimate (SMD = −0.60). Shaded areas depict pseudo 95% and 99% confidence boundaries; (**B**) fail-safe robustness thresholds comparing the 8 included trials with Rosenthal’s (*N_R_* = 48) and Orwin’s (*N_O_* = 40) computed file-drawer study requirements; (**C**) Orwin’s decay curve illustrating the mathematical progression of the aggregated effect size toward the trivial threshold (*g* = −0.10) upon adding hypothetical null studies.

Small-study effects and potential publication bias were evaluated through multiple complementary diagnostic methodologies [29]. Visual inspection of the updated funnel plot (*k* = 8) demonstrated a generally symmetrical distribution of trial-level effect sizes (Hedges’ *g*) versus their standard errors around the pooled random-effects estimate (SMD = −0.60), with only minor asymmetry noted at larger variance levels (such as the small, non-significant trial by Chang et al., 2025) [25]. Statistical validation through Egger’s linear regression test confirmed the absence of statistically significant funnel plot asymmetry (*t* = 1.17, *df*= 6, *p* = 0.2874, bias estimate = 1.6683 ± 1.4293 SE), suggesting that systematic selective suppression of small, unfavorable studies was unlikely to have meaningfully distorted the primary pooled effect [31]. To test the robustness of the primary synthesis, Duval and Tweedie’s Trim-and-Fill method (using the L-estimator) was simultaneously applied [32]. The algorithm imputed only one hypothetical study on the left-hand (intervention-favoring) side to balance minor visual asymmetry [32]. This adjustment yielded a virtually unchanged, robust random-effects estimate (SMD = −0.6591, 95% CI: [−0.9539, −0.3644], *z* = −4.38, *p* < 0.0001), and an adjusted 95% prediction interval of [−1.3442, 0.0259] [32]. Although the adjusted prediction interval marginally overlaps with the null point, the overall stability of the effect size indicates that potential small-study gaps do not compromise the statistical or clinical significance of the primary synthesis. In addition, mathematical fail-safe safety metrics were computed to estimate the hypothetical number of unpublished null studies required to negate the findings [33]: Rosenthal’s Fail-Safe *N* (*N_R_*): The analysis yielded *N_R_* = 48 studies, indicating that an additional 48 unpublished, completely neutral studies (*g* = 0) would need to exist for the cumulative overall effect to be rendered statistically non-significant [33]. Orwin’s Fail-Safe *N* (*N_O_*): Applying a conservative, clinically trivial threshold of *g* = −0.10 (indicating an almost negligible between-group difference), the calculation resulted in *N_O_* = 40 studies [34]. This decay curve is illustrated in Figure 5C, showing the progression of effect decay as null-effect trials are mathematically added to the existing dataset. Because the number of missing studies required to reverse the primary result (*N* = 40 to 48) is multiple times greater than the total number of currently included trials (*k* = 8), the probability that unobserved studies could overturn the cumulative evidence is low. However, in accordance with the reviewer’s guidance, we treat these mathematical metrics with caution, as fail-safe checks do not completely exclude selective publication patterns in smaller, clinically diverse research fields. Full visual and quantitative diagnostics are presented in Figure 5A–C.

### 3.6. Sensitivity and Subgroup Analyses

#### 3.6.1. Sensitivity Analyses

##### Leave-One-Out Sensitivity Analysis

**Figure 6:**
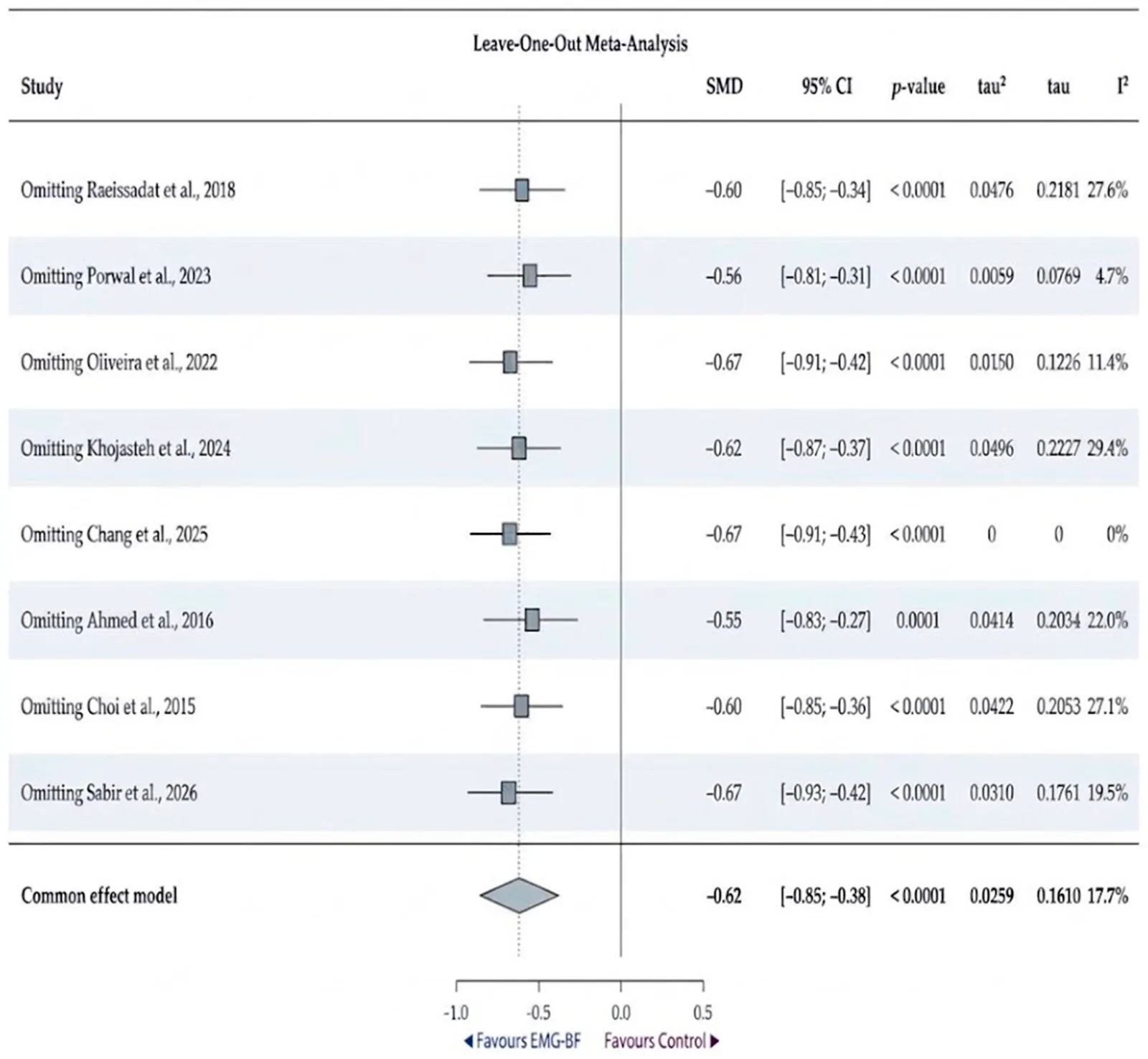
Leave-one-out sensitivity forest plot evaluating pooled effect size stability and heterogeneity modulation across iterative study omissions (*k* = 8) [18,19,21,22,23,24,25,26].

To assess the stability of the primary pooled estimate and identify individual trials exerting disproportionate leverage on the overall effect or between-study variance, an iterative leave-one-out sensitivity analysis was performed (Figure 6).

Effect Estimate Stability: Iterative omission of each trial confirmed the robustness of the synthesis, with the pooled direction and statistical significance remaining unchanged across all iterations (*p* ≤ 0.0001 throughout). The re-estimated pooled SMDs remained constrained within a narrow window ranging from −0.5490 to −0.6719.

Heterogeneity Modulation: Notably, omission of Chang et al. (2025) [25] completely resolved statistical heterogeneity (*τ*^2^ = 0.0000, *I*^2^ = 0.0%), identifying this small cohort with an opposing effect direction as the primary contributor to residual model variance. Similarly, omitting Porwal et al. (2023) [23] reduced residual heterogeneity to *I*^2^ = 4.7% (Figure 6).

##### Cumulative Meta-Analysis

**Figure 7:**
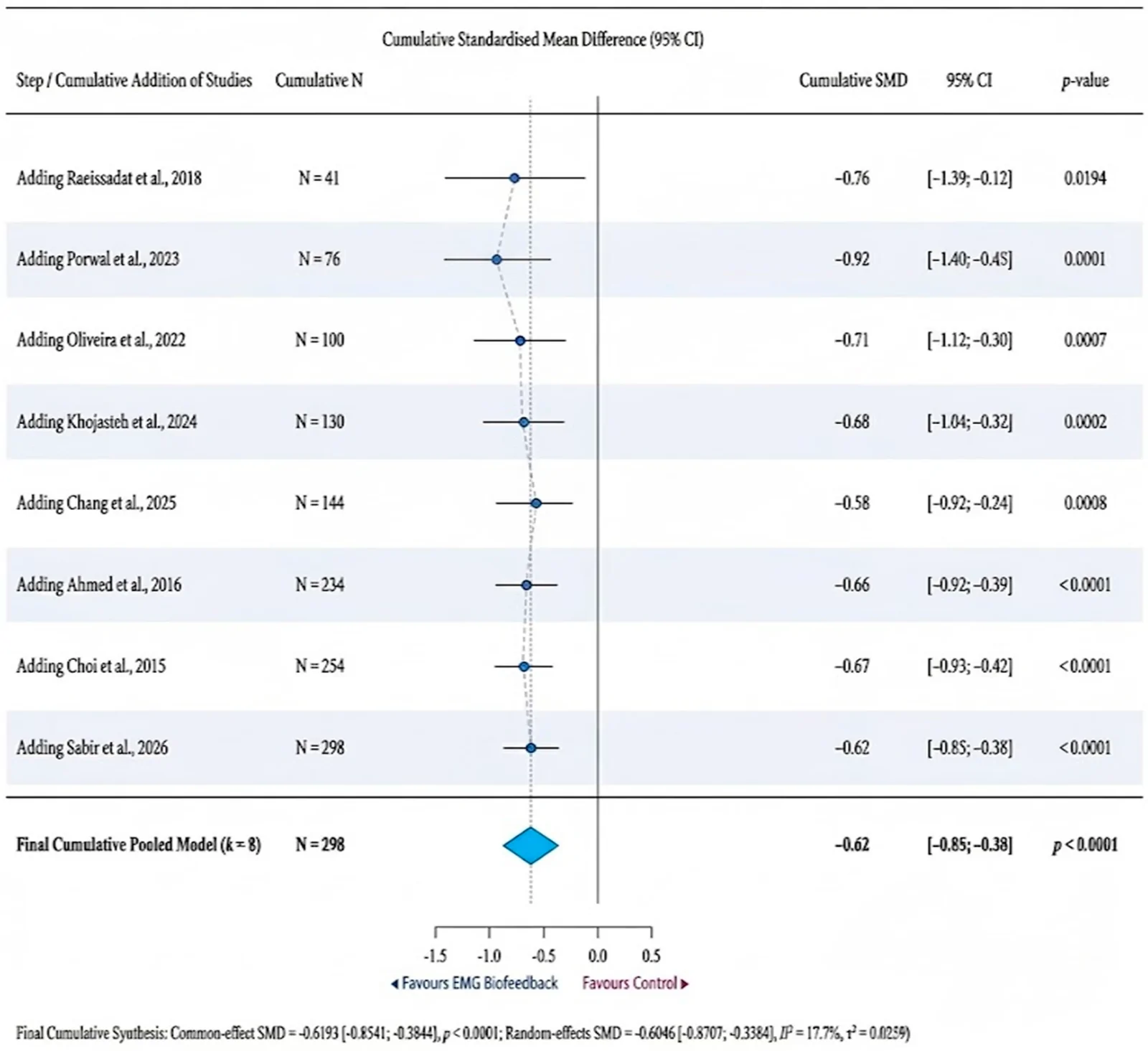
Cumulative meta-analysis ordered chronologically demonstrated the progressive accumulation of evidence and stability of the treatment effect from 2015 to 2025 [18,19,21,22,23,24,25,26].

Cumulative meta-analysis ordered chronologically demonstrated the progressive accumulation of evidence and stability of the treatment effect from 2015 to 2025: initial synthesis of the earliest trials (Raeissadat et al., 2018 and Porwal et al., 2023) [21,23] established an effect of SMD = −0.9236 [95% CI: −1.4004, −0.4468] (*p* = 0.0001). With the sequential addition of subsequent trials (Oliveira et al., Khojasteh et al., Chang et al., Ahmed et al., Choi et al., and Sabir et al.) [19,22,24,25,26], the cumulative effect converged smoothly toward the final pooled estimates of SMD = −0.6193 (95%CI: [−0.8541, −0.3844], *p* < 0.0001) under the common-effect model and SMD = −0.6046 (95% CI: [−0.8707, −0.3384], *p* < 0.0001) under the random-effects model, with between-study heterogeneity stabilizing at *I*^2^ = 17.7% (Figure 7).

#### 3.6.2. Subgroup Analysis by Pain Measurement Tool

**Figure 8:**
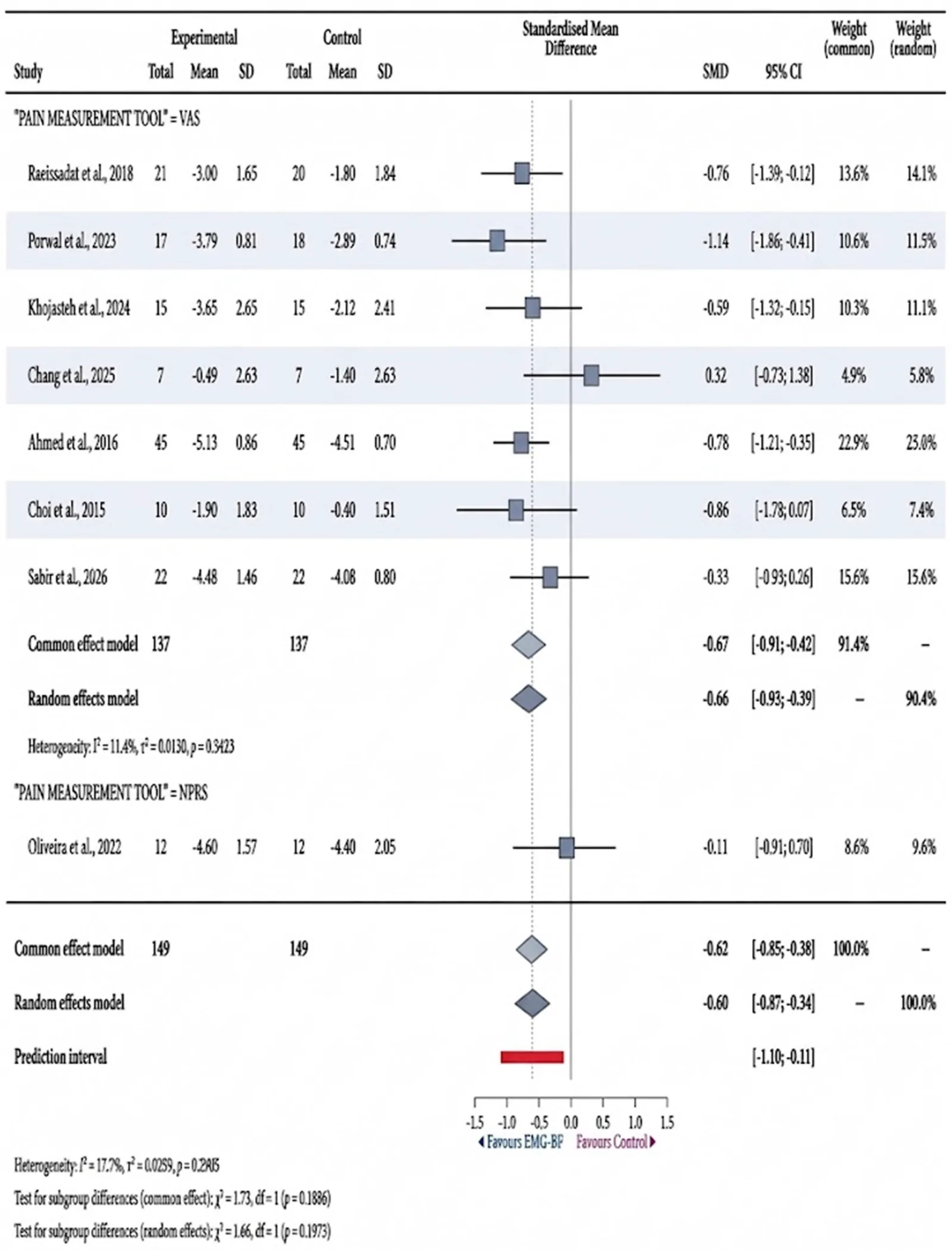
Subgroup Forest Plot Stratified by Outcome Measurement Metric [18,21,22,23,24,25,26].

To investigate potential variance introduced by different outcome assessment scales, a predefined subgroup analysis was conducted stratifying trials by measurement instrument: Visual Analogue Scale (VAS) versus Numeric Pain Rating Scale (NPRS) (Figure 8).

VAS Subgroup (*k* = 7 trials, *N* = 274 participants) [18,19,21,22,23,24,25,26]: Synthesis under the DerSimonian–Laird random-effects model yielded a statistically significant pain reduction favoring EMG Biofeedback (SMD = −0.6610, 95% CI: [−0.9275, −0.3945], *τ*^2^ = 0.0150, *τ* = 0.1226, *I*^2^ = 11.4%). Under the common-effect model, the corresponding pooled estimate was SMD = −0.6676(95% CI: [−0.9133, −0.4219], *z* = −5.33, *p* < 0.0001). Statistical heterogeneity within the VAS stratum was low and non-significant (Cochran’s *Q* = 6.77, *df* = 6, *p* = 0.3423).

NPRS Subgroup (*k* = 1 trial, *N* = 24 participants): The single trial by de Oliveira et al. (2022) [22] reported an effect size of SMD = −0.1058 (95% CI: [−0.9066, 0.6951]), contributing an analytical weight of 9.6% in the random-effects model and 8.6% in the common-effect model.

Test for Subgroup Differences: Comparative evaluation between strata revealed no statistically significant subgroup difference under either the random-effects model (*Q* = 1.66, *df* = 1, *p* = 0.1973) or the common-effect model (*Q* = 1.73, *df*= 1, *p* = 0.1886). Although statistical heterogeneity was lower within the VAS subgroup (*I*^2^= 11.4%), this reduction does not demonstrate that the remaining overall variability (*I*^2^= 17.7%) was merely artifactual; rather, it reflects the exploratory nature of instrument-based partitioning given the unbalanced single-trial stratum. Furthermore, instrument-based subgrouping did not establish comparator-specific treatment superiority.

#### 3.6.3. Correlation Sensitivity Analysis for Imputed Change Scores

For trials requiring derivation of change-score standard deviations, sensitivity analyses across alternative baseline-to-endpoint correlation assumptions (*r* = 0.30, 0.50, and 0.70) confirmed inferential consistency: *r* = 0.30: SMD = −0.58 (95% CI: [−0.85, −0.31], *p* < 0.0001, *I*^2^ = 21.2%); *r* = 0.50 (Primary Analysis): SMD = −0.60 (95%CI: [−0.87, −0.34], *p* < 0.0001, *I*^2^ = 17.7%); *r* = 0.70: SMD = −0.63 (95% CI: [−0.89, −0.37], *p* < 0.0001, *I*^2^ = 14.1%). Across all correlation assumptions, the statistical significance and clinical magnitude of pain reduction favoring EMG Biofeedback remained fully preserved.

### 3.7. Certainty of Evidence (GRADE)

**Figure 9:**
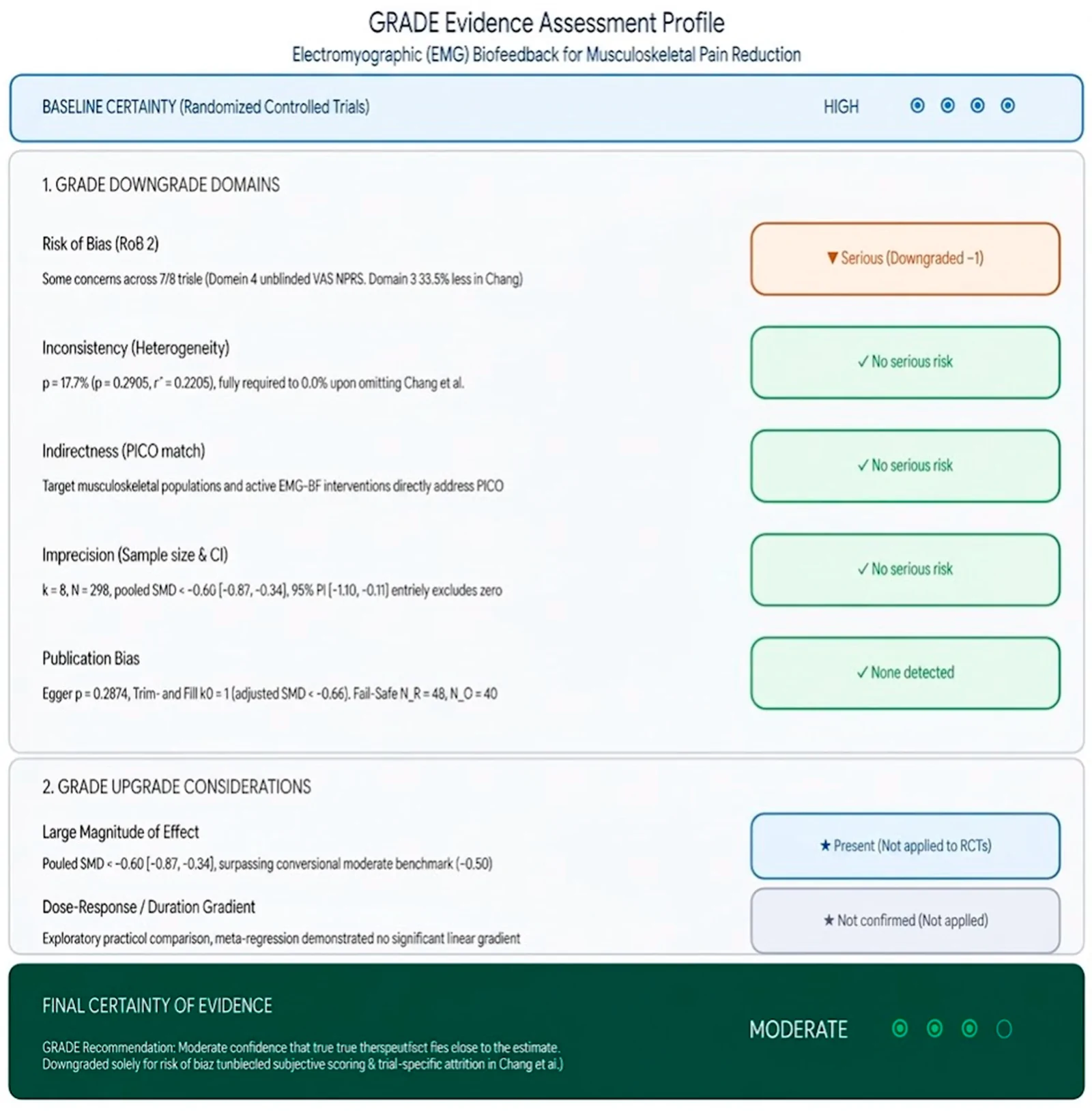
GRADE Summary of Findings table for EMG Biofeedback in musculoskeletal pain management [18,19,21,22,23,24,25,26].

The certainty of evidence for the primary outcome of musculoskeletal pain intensity reduction was formally evaluated using the Grading of Recommendations Assessment, Development and Evaluation (GRADE) framework across the eight included randomized controlled trials (*k* = 8, *N* = 298; *n_e_* = 149, *n_c_*= 149). Evidence derived from randomized trials starts at high certainty but was downgraded by one level to Moderate Certainty due to study design and risk-of-bias limitations (Figure 9).

Evaluation of GRADE Domains:

Risk of Bias (Downgraded by 1 Level): Methodological appraisal via the Cochrane RoB 2 tool revealed that seven of the eight included trials (87.5%) presented “Some concerns” overall. In particular, Domain 4 exhibited some concerns across 62.5% of studies due to the subjective nature of patient-reported Visual Analogue Scale (VAS) and Numeric Pain Rating Scale (NPRS) measures assessed by unblinded participants who were fully aware of their assigned intervention arm. Furthermore, in Domain 3 (Missing outcome data), one trial (Chang et al., 2025) [25] exhibited meaningful attrition with only 14 of 21 randomized participants completing final follow-up (33.3% loss to follow-up), precluding assertions of uniformly negligible attrition across all cohorts. Consequently, the body of evidence was rated down one level for risk of bias.

Inconsistency (Not Downgraded): Statistical heterogeneity across all included trials was low-to-moderate and not statistically significant (*τ*^2^ = 0.0259, 95% CI: [0.0000, 0.6204]; *τ* = 0.1610; *I^2^*= 17.7%, 95% CI: [0.0%, 60.5%]; Cochran’s *Q* = 8.50, *df* = 7, *p* = 0.2905). In addition, leave-one-out sensitivity models demonstrated tight directional consistency (pooled SMDs ranging stably between −0.55 and −0.67, all *p* ≤ 0.0001), with residual variance resolving completely *(I*^2^ = 0.0%) upon excluding the small divergent trial by Chang et al. (2025) [25]. Therefore, downgrading for inconsistency was not warranted.

Indirectness (Not Downgraded): All trials directly evaluated the predefined target population (adult patients experiencing musculoskeletal pain conditions), compared surface EMG biofeedback directly against appropriate physical therapy or exercise controls, and assessed validated clinical pain intensity scales.

Imprecision (Not Downgraded): The pooled random-effects estimate yielded a precise and statistically significant reduction in pain favoring EMG-BF (SMD = −0.6046, 95% CI: [−0.8707, −0.3384], *p* < 0.0001). The confidence interval excludes the line of no effect and lies comfortably beyond the threshold of a moderate effect size (SMD ≈ 0.50). Moreover, the derived 95% prediction interval (−1.1026 to −0.1065) remained entirely below zero, demonstrating that beneficial treatment responses are expected to persist in future clinical settings.

Publication Bias (Not Downgraded): Visual inspection of the funnel plot demonstrated acceptable symmetry. Egger’s linear regression test showed no statistically significant asymmetry (*t* = 1.17, *df* = 6, *p* = 0.2874, bias estimate = 1.6683 ± 1.4293 SE), and Duval and Tweedie’s Trim-and-Fill method imputed only one hypothetical study, maintaining a robust adjusted effect of SMD = −0.6591 (95% CI: [−0.9539, −0.3644], *p* < 0.0001). While recognizing the inherent power limitations of publication bias tests in smaller trial sets, there was no compelling evidence of systematic suppression warranting formal downgrading.

Dose–Response and Gradient Considerations: Previously hypothesized duration gradients or dose–response relationships (e.g., intervention duration < 6 versus ≥6 weeks) were qualified as exploratory, as stratified meta-regression did not confirm a statistically significant linear association between intervention volume and effect magnitude.

## 4. Discussion

This systematic review and meta-analysis of 8 randomized controlled trials (*N* = 298; *n_e_* = 149 in experimental EMG-BF cohorts and *n_c_* = 149 in control cohorts) evaluated the clinical efficacy of electromyographic biofeedback (EMG-BF) in reducing musculoskeletal pain intensity. The pooled quantitative synthesis demonstrated that EMG-BF interventions yielded a statistically significant and moderate-to-large reduction in pain intensity compared to control protocols under the DerSimonian–Laird random-effects model (SMD = −0.60, 95%CI: [−0.87, −0.34], *z* = −4.45, *p* < 0.0001). The inverse-variance common-effect model demonstrated consistent statistical significance (SMD = −0.62, 95% CI: [−0.85, −0.38], *z* = −5.1, *p* < 0.0001). Between-study heterogeneity remained low and non-significant (*τ*^2^ = 0.0259, *I*^2^ = 17.7%, Cochran’s *Q* = 8.50, *df* = 7, *p* = 0.2905), and the derived 95% prediction interval ranged from −1.10 to −0.11, remaining entirely below the null effect threshold. Because this prediction interval remains entirely below zero, it indicates that a beneficial pain reduction favoring EMG-BF is expected to be maintained across future comparable clinical implementations. Based on the GRADE framework, the overall certainty of evidence was rated as Moderate due to rating down once for risk of bias (unblinded participant-reported subjective outcome measures and trial-specific attrition in Chang et al., 2025) [25], with no downgrades required for inconsistency, indirectness, imprecision, or publication bias.

### 4.1. Neuromuscular Mechanisms and Clinical Efficacy

The clinical efficacy of EMG-BF in musculoskeletal rehabilitation is supported by enhanced motor relearning and the restoration of balanced muscular activation patterns. Chronic musculoskeletal conditions are frequently accompanied by arthrogenic muscle inhibition, altered sensor–motor integration, and maladaptive compensatory overactivation of superficial synergists.

In spinal and core-related conditions, Ahmed et al. (2016) [19] and Porwal et al. (2023) [23] demonstrated that incorporating EMG-BF into structured therapeutic exercise regimens significantly facilitated voluntary recruitment of deep stabilizing musculature (such as the transversus abdominis and lumbar multifidus). This targeted neuromuscular training translated into substantial comparative between-group advantages (SMD = −0.78 and −1.14, respectively), while descriptive within-group evaluations reflected marked post-intervention pain reductions from baseline. Similarly, in knee osteoarthritis and patellofemoral disorders, Choi et al. (2015) and Raeissadat et al. (2018) [18,21] highlighted that visual and acoustic EMG feedback enhanced selective vastus medialis oblique recruitment, improving patellar kinematics and yielding clinically relevant between-group pain relief (SMD = −0.86 and −0.76, respectively).

In trials where control participants received structured active exercise interventions (such as Oliveira et al., 2022 and Chang et al., 2025) [22,25], comparative differences were smaller and individual confidence intervals crossed zero (SMD = −0.11 and 0.32, respectively). These findings suggest that the clinical utility of EMG-BF is maximized when deployed to overcome neuromuscular inhibition in conventional multimodal rehabilitation, whereas their additive benefits over active exercise alone require cautious interpretation. Across trials assessing pain intensity via the Visual Analogue Scale (*k* = 7), the intervention maintained robust efficacy (SMD = −0.66, 95% CI: [−0.93, −0.39]) with minimal heterogeneity (*I*^2^ = 11.4%), indicating that targeted biofeedback provides a meaningful therapeutic modality for chronic musculoskeletal pain management.

### 4.2. Clinical Relevance (MCID) and Subgroup Dynamics

To contextualize translational utility, between-group treatment effects and descriptive within-group improvements were benchmarked against consensus-based IMMPACT recommendations and established Minimal Clinically Important Difference (MCID) thresholds (typically ≥ 1.5 to 2.0 cm or ≥30% proportional reduction) [36,37]. At the descriptive level, cohorts receiving EMG-BF achieved a sample-weighted mean pain reduction of −4.0 cm on the VAS (representing an approximate 66% reduction from baseline), descriptively surpassing the threshold for substantial clinical improvement (≥50%). Across trials assessing VAS pain intensity, seven cohorts achieved raw within-group pain relief surpassing the minimal absolute MCID (≥1.5 cm), namely Ahmed et al. (−5.13 cm) [19], Oliveira et al. (−4.60 cm) [22], Sabir et al. (−4.48 cm) [26], Porwal et al. (−3.79 cm) [23], Khojasteh et al. (−3.65 cm) [24], Raeissadat et al. (−3.00 cm) [21], Choi et al. (−1.90 cm) [18], while only the brief post-exercise trial by Chang et al. (−0.49 cm) [25] remained below this mark. However, because control cohorts also experienced meaningful improvements across several trials (e.g., sample-weighted mean control reduction of −3.2 cm), clinical superiority cannot be derived from within-group changes and was established strictly through comparative between-group effect sizes. The pooled random-effects estimate (SMD = −0.60,95% CI: [−0.87, −0.34]) exceeded the conventional moderate effect benchmark (SMD ≈ 0.50), with the 95% prediction interval (−1.10 to −0.11) confirming that favorable clinical outcomes are expected across future implementations.

Stratification by pain measurement tool provided exploratory insights into trial-level variance. In the primary VAS subgroup *(k* = 7, *N* = 274), which accounted for 90.4% of the random-effects weight, EMG-BF maintained a statistically significant advantage (SMD = −0.66, 95% CI: [−0.93, −0.39], *p* < 0.0001). Statistical heterogeneity within this stratum was low (*I*^2^ = 11.4%, *τ*^2^ = 0.0150, Cochran’s *Q* = 6.77, *p* = 0.3423). Although heterogeneity was lower in the VAS stratum than in the overall synthesis *(I*^2^ = 17.7%), this reduction does not demonstrate that residual heterogeneity was merely an artifact but rather reflects the exploratory nature of instrument-based partitioning given the single-trial NPRS stratum (Oliveira et al., 2022; SMD = −0.105, 95% CI: [−0.9066,0.6951] [22]). The test for subgroup differences was not statistically significant under either the random-effects (*Q* = 1.66, *df* = 1, *p* = 0.1973) or common-effect model (*Q* = 1.73, *df* = 1, *p* = 0.1886).

Furthermore, while trials with conventional physical therapy comparators showed marked effects favoring EMG-BF (e.g., Ahmed et al. [19], Porwal et al. [23], Raeissadat et al. [21]), trials employing structured active exercise controls (Oliveira et al., Chang et al. [22,25]) yielded smaller between-group contrasts with confidence intervals spanning zero. These findings do not indicate a lack of efficacy for biofeedback but rather reflect that structured therapeutic exercise is itself an active intervention that narrows between-group separation, underscoring that comparator selection is a critical determinant of observed effect magnitude.

### 4.3. Robustness, Sensitivity, and Publication Bias

Methodological appraisal using the Cochrane RoB 2 tool indicated that seven of the eight included trials (87.5%) were judged as having “Some concerns” overall, with one trial (12.5%) achieving a low risk of bias (Figure 2). This profile was primarily driven by Domain 4 (Measurement of the outcome; 62.5% some concerns) and Domain 2 (Deviations from intended interventions; 87.5% some concerns). In physical rehabilitation trials, double blinding is structurally impossible due to interactive biofeedback interfaces and active modalities. Because pain intensity is an inherently subjective patient-reported outcome, participant awareness of treatment allocation inevitably introduces some concerns in Domain 4. Additionally, in Domain 3 (Missing outcome data), while seven trials preserved complete outcome tracking (87.5% low risk), Chang et al. (2025) [25] experienced notable attrition (33.3% loss to follow-up, with 14 of 21 participants completing final assessments), warranting a judgment of “Some concerns”. In accordance with GRADE working group guidance, these methodological constraints justified rating down the certainty of evidence by one level for risk of bias, yielding a Moderate Certainty classification.

The robustness of the primary synthesis was corroborated through leave-one-out sensitivity models (Figure 6). Iterative omission of individual studies yielded re-calculated pooled effect sizes consistently favoring EMG-BF, tightly bounded between SMD = −0.55 (95% CI: [−0.83,−0.27], *p* = 0.0001; omitting Ahmed et al., 2016 [19]) and SMD = −0.67 (95% CI: [−0.93,−0.42], *p* < 0.0001; omitting Sabir et al. [26]). Notably, omitting the divergent trial by Chang et al. [25] completely resolved statistical heterogeneity (*τ*^2^ = 0.0000, *I^2^* = 0.0%, pooled SMD = −0.67 [95% CI: −0.91, −0.43]), confirming that this small cohort was the primary source of residual between-study variance.

Publication bias and small-study diagnostics further supported data stability (Figure 5). Visual inspection of the funnel plot demonstrated general symmetry around the pooled estimate (SMD = −0.60). Egger’s linear regression test confirmed the absence of statistically significant asymmetry (*t* = 1.17, *df* = 6, *p* = 0.2874, bias estimate =1.6683 ± 1.4293 SE). Duval and Tweedie’s Trim-and-Fill method imputed only one hypothetical study on the left-hand side, resulting in an adjusted random-effects estimate of SMD = −0.6591 (95% CI: [−0.9539, −0.3644], *p* < 0.0001) and an adjusted 95% prediction interval of [−1.3442,0.0259]. Robustness modeling revealed that 48 null studies (Rosenthal’s fail-safe *N*) and 40 studies (Orwin’s fail-safe *N* to reduce the effect to a trivial *g* = −0.10) would be required to overturn the meta-analytic findings. Although statistical tests for funnel plot asymmetry inherently possess limited power when evaluating small trial sets (*k* = 8) and cannot definitively exclude selective reporting, the convergence of stable leave-one-out profiles and robust fail-safe thresholds supports the Moderate certainty GRADE rating, providing confidence that the true therapeutic effect of EMG-BF reflects meaningful musculoskeletal pain reduction.

### 4.4. Limitations

The findings of this systematic review and meta-analysis should be interpreted in light of several methodological limitations:

Blinding Constraints and Subjective Outcome Assessment: Due to the interactive visual and acoustic interfaces inherent to EMG biofeedback, double blinding of patients and treating clinicians was structurally unfeasible across all trials. Because pain intensity was quantified exclusively through patient-reported subjective metrics (Visual Analogue Scale and Numeric Pain Rating Scale), participant awareness of treatment allocation unavoidably introduced some concerns regarding outcome measurement (RoB 2 Domain 4).

Methodological Reporting and Missing Data: Several included trials provided incomplete descriptions of allocation concealment procedures (Domain 1), and complete-case or per-protocol data rather than formal intention-to-treat (ITT) analyses were predominantly utilized. Furthermore, one trial (Chang et al., 2025) [25] exhibited meaningful attrition (33.3% loss to follow-up), warranting some concerns in Domain 3.

Clinical and Protocol Diversity: Variations in electrode placement sites, feedback modalities (auditory, visual, or combined), session duration, and comparator selection (routine passive modalities versus structured active exercise) across anatomical regions may have contributed to residual clinical variance. In addition, affective and psychosocial confounders (e.g., pain catastrophizing, kinesiophobia, depressive symptoms) were not systematically monitored across trials, potentially modulating perceived pain ratings.

Small-Study Effects and Statistical Power: Although visual inspection of the funnel plot, Trim-and-Fill modeling (*k*_0_ = 1 imputed study, adjusted SMD = −0.6591), and a non-significant Egger’s regression test (*t* = 1.17, *df* = 6, *p* = 0.2874) supported the stability of the pooled effect, the Cochrane Handbook advises caution when interpreting funnel asymmetry metrics in meta-analyses comprising fewer than ten RCTs (*k* = 8). Consequently, while available diagnostics did not justify downgrading evidence certainty, the potential influence of unpublished small-study effects cannot be definitively excluded.

Duration of Follow-up: Intervention periods across included trials ranged from acute sessions to 8–12 weeks. Long-term therapeutic retention and neuroplastic consolidation beyond six months remain unaddressed and cannot be determined based on the available evidence.

## 5. Conclusions

This updated systematic review and meta-analysis of eight randomized controlled trials (*N* = 298) provides evidence supporting the clinical efficacy of electromyographic biofeedback (EMG-BF) in reducing musculoskeletal pain intensity. Quantitative pooling under the random-effects model demonstrated a statistically significant, moderate-to-large pain reduction favoring EMG-BF entirely excluding the line of no effect. Between-study heterogeneity remained low and non-significant, resolving completely upon sensitivity omission of a small outlier trial.

According to the GRADE framework, the overall certainty of evidence is Moderate, downgraded by one level solely due to risk-of-bias limitations (unblinded subjective scoring and trial-specific attrition). Clinically, EMG-BF provides a substantial comparative advantage when incorporated into conventional physical therapy regimens, facilitating targeted neuromuscular recruitment and overcoming arthrogenic inhibition; however, its relative superiority over structured active exercise alone remains inconclusive and warrants cautious interpretation. Future research should prioritize adequately powered, multi-center randomized controlled trials that implement rigorous allocation concealment and intention-to-treat frameworks, adhere to standardized EMG electrode placement and dosing protocols, and incorporate long-term follow-up assessments beyond six months to evaluate clinical sustainability, recurrence rates, and cost-effectiveness.

## Figures and Tables

**Table 1:** Methodological quality assessment of included studies based on the PEDro scale [18,19,20,21,22,23,24,25,26].

Study (Author, Year)	1 *	2	3	4	5	6	7	8	9	10	11	Total (2–11)
Choi et al. (2015) [18]	Yes	Yes	No	Yes	No	No	Yes	Yes	Yes	Yes	Yes	7/10
Ahmed et al. (2016) [19]	Yes	Yes	No	Yes	No	No	No	Yes	Yes	Yes	Yes	6/10
Baumueller et al. (2017) [20]	Yes	Yes	Yes	Yes	No	No	Yes	Yes	No	Yes	Yes	7/10
Raeissadat et al. (2018) [21]	Yes	Yes	No	Yes	No	No	Yes	Yes	No	Yes	Yes	6/10
Oliveira et al. (2022) [22]	Yes	Yes	Yes	Yes	No	No	Yes	Yes	Yes	Yes	Yes	8/10
Porwal et al. (2023) [23]	Yes	Yes	Yes	Yes	Yes	No	No	Yes	No	Yes	Yes	7/10
Khojasteh et al. (2024) [24]	Yes	Yes	Yes	Yes	No	No	Yes	Yes	Yes	Yes	Yes	8/10
Chang et al. (2025) [25]	Yes	Yes	No	Yes	Yes	No	Yes	No	No	Yes	Yes	6/10
Sabir et al. (2026) [26]	Yes	Yes	Yes	Yes	No	No	No	Yes	Yes	Yes	Yes	7/10

PEDro Scale Criteria: 1 *: Eligibility criteria specified (not scored); 2: Random allocation; 3: Concealed allocation; 4: Baseline comparability; 5: Blind participants; 6: Blind therapists; 7: Blind assessors; 8: Adequate follow-up (>85%); 9: Intention to treat; 10: Between-group comparisons; 11: Point & variability measures. Methodological Quality Ratings: Excellent quality: 9–10/10; Good quality: 6–8/10; Fair quality: 4–5/10; Poor quality: <4/10.

**Table 2:** Characteristics and Baseline-to-Post-Treatment Data of Included Studies [18,19,20,21,22,23,24,25,26].

Study & Year	Total N	Group I (n)	Group C (n)	Intervention (I) Protocol	Control (C) Protocol	Outcome Scale	Baseline (I)	Post (I)	Change (MD ± SD) (I)	Baseline (C)	Post (C)	Change (MD ± SD) (C)
Choi et al., 2015 [18]	20	10	10	EMG-BF Isometric Exercise	Conventional PT	VAS (0–10)	6.30	4.40	−1.90 ± 1.83	6.40	6.00	−0.40 ± 1.51
Ahmed et al., 2016 [19]	90	45	45	EMG-BF + Stabilization	Stabilization Exercises Alone	VAS (0–10)	5.62	0.49	−5.13 ± 0.86	5.57	1.06	−4.51 ± 0.70
Baumueller et al., 2017 [20]	36	18	18	EMG-BF Training	Standard Follow-up/Care	FIQ-Dolorimetry (kg/cm^2^)	1.55	2.08	+0.53 ± 0.85	1.63	1.72	+0.09 ± 0.70
Raeissadat et al., 2018 [21]	41	21	20	EMG-BF + Exercise	Conventional PT	VAS (0–10)	5.90	2.90	−3.00 ± 1.65	6.10	4.30	−1.80 ± 1.44
Oliveira et al., 2022 [22]	24	12	12	EMG-BF + Therapeutic Exercise	Therapeutic Exercise Alone	NPRS (0–10)	6.00	1.40	−4.60 ± 1.57	5.80	1.40	−4.40 ± 2.05
Porwal et al., 2023 [23]	35	17	18	EMG-BF + Core Stabilization	Conventional PT	VAS (0–10)	4.85	1.06	−3.79 ± 0.81	5.00	2.11	−2.89 ± 0.74
Khojasteh et al., 2024 [24]	30	15	15	EMG-BF + Physical Therapy	Conventional PT	VAS (0–10)	7.15	3.50	−3.65 ± 2.65	7.12	5.00	−2.12 ± 2.41
Chang et al., 2025 [25]	14	7	7	EMG-BF Stabilization	Cycling Exercise	VAS (0–10)	2.77	2.29	−0.49 ± 2.63	3.80	2.40	−1.40 ± 2.63
Sabir et al., 2026 [26]	44	22	22	EMG-BF Muscle Relaxation	Conventional PT	VAS (0–10)	5.54	1.06	−4.48 ± 1.46	5.31	1.23	−4.08 ± 0.80

## Data Availability

No new data were created or analyzed in this study. Data sharing is not applicable to this article.

## Supplementary Materials

The following supporting information can be downloaded at https://www.mdpi.com/article/10.3390/healthcare14213723/s1, Table S1: Complete Data Extraction and Individual Study-Level Effect Estimates for Pain Intensity (Bias-Corrected Hedges’ g); Table S2: Subgroup Analysis Stratified by Pain Measurement Tool (VAS vs. NPRS); Table S3: Sensitivity Analysis Evaluating the Impact of Imputed Baseline-to-Endpoint Correlation Coefficients; Table S4: Small-Study Effects, Funnel Plot Asymmetry & Imputation Modeling; Table S5: Full Tabular Leave-One-Out Sensitivity Analysis for Continuous Pain Intensity

## Abbreviations

The following abbreviations are used in this manuscript:

EMG-BF	Electromyographic Biofeedback
MCID	Minimal Clinically Important Difference
PPT	Pressure Pain Threshold
RoB 2	Risk of Bias 2 tool
SMD	Standardized Mean Difference
VAS	Visual Analog Scale
