# Supplementary material for: The Effectiveness of Electromyographic Biofeedback in Reducing Musculoskeletal Pain: A Systematic Review and Meta-Analysis
Source: Healthcare (Basel). 2026 Nov 2;14(21):3723. doi: 10.3390/healthcare14213723 (PMC13762655; doi:10.3390/healthcare14213723)
Supplement: Supplementary file 1 [file healthcare-14-03723-s001.zip › healthcare-4583652-supplementary.pdf]

# Table S1 (Section B): Complete Data Extraction & Individual Study Effect Sizes

## EMG-BFB for Musculoskeletal Pain Full Synthesis ($k$ = 8, $N$ = 298). Hedges' $g$ (DerSimonian-Laird)

All statistical analysis was performed with “The jamovi project (2025). *jamovi*. (Version 2.7) [Computer software]. Retrieved from https://www.jamovi.org”.

**Section A: Cochrane Handbook Formula for Change-Score Standard Deviation ($SD_{change}$):** $SD_{change} = \sqrt{[SD_{baseline}^2 + SD_{final}^2 - (2 \times r \times SD_{baseline} \times SD_{final})]}$. Imputed with baseline correlation $r$ = 0.50 and verified across sensitivity intervals ($r$ = 0.30 to 0.70).

**Table S1 (Section B). Full Extracted Numerical Dataset, Standardized Mean Differences (SMD), 95% Confidence Intervals & Exact Model Weights.**

| STUDY CHARACTERISTICS | | EMG-BFB (EXPERIMENTAL) | | | CONTROL COMPARATOR | | | EFFECT SIZE (HEDGES' G) | | MODEL WEIGHTS | |
|---|---|---|---|---|---|---|---|---|---|---|---|
| Study Citation | Tool | Mean | SD | n | Mean | SD | n | SMD (g) | 95% Confidence Interval | % W (Common) | % W (Random) |
| Raeissadat et al., 2018 | VAS | -3.00 | 1.65 | 21 | -1.80 | 1.44 | 20 | -0.7586 | [-1.3947; -0.1224] | 13.6% | 14.1% |
| Porwal et al., 2023 | VAS | -3.79 | 0.81 | 17 | -2.89 | 0.74 | 18 | -1.1351 | [-1.8552; -0.4150] | 10.6% | 11.5% |
| Oliveira et al., 2022 | NPRS | -4.60 | 1.57 | 12 | -4.40 | 2.05 | 12 | -0.1058 | [-0.9066; 0.6951] | 8.6% | 9.6% |
| Khojasteh et al., 2024 | VAS | -3.65 | 2.65 | 15 | -2.12 | 2.41 | 15 | -0.5877 | [-1.3208; 0.1454] | 10.3% | 11.1% |
| Chang et al., 2025 | VAS | -0.49 | 2.63 | 7 | -1.40 | 2.63 | 7 | 0.3238 | [-0.7331; 1.3808] | 4.9% | 5.8% |
| Ahmed et al., 2016 | VAS | -5.13 | 0.86 | 45 | -4.51 | 0.70 | 45 | -0.7840 | [-1.2134; -0.3545] | 29.9% | 25.0% |
| Choi et al., 2015 | VAS | -1.90 | 1.83 | 10 | -0.40 | 1.51 | 10 | -0.8562 | [-1.7808; 0.0684] | 6.5% | 7.4% |
| Sabir et al., 2016 | VAS | -4.48 | 1.46 | 22 | -4.08 | 0.80 | 22 | -0.3337 | [-0.9291; 0.2618] | 15.6% | 15.6% |
| **Common Effect Model (Fixed Effect)** | | | | **149** | | | **149** | **-0.6193** | **[-0.8541; -0.3844]** | **100.0%** | **—** |
| **Random Effects Model (DerSimonian-Laird)** | | | | **149** | | | **149** | **-0.6046** | **[-0.8707; -0.3384]** | **—** | **100.0%** |
| **95% Prediction Interval (Student's $t$, $df$ = 7)** | | | | **149** | | | **149** | | **[-1.1026; -0.1065]** | **—** | **—** |

**Section B Statistical Metrics (Complete):** Number of studies: $k$ = 8. Total observations: $N$ = 298 ($n_e$ = 149 experimental, $n_c$ = 149 control). Common effect test for overall effect: $z$ = -5.17, $p$ < 0.0001. Random effects test for overall effect: $z$ = -4.45, $p$ < 0.0001. 95% Prediction Interval: [-1.1026; -0.1065]. Heterogeneity: $\tau^2$ = 0.0259 [95% CI: 0.0000; 0.6204]; $\tau$ = 0.1610 [95% CI: 0.0000; 0.7876]; $I^2$ = 17.7% [95% CI: 0.0%; 60.5%]; $H$ = 1.10 [95% CI: 1.00; 1.59]. Cochran's heterogeneity test: $Q$ = 8.50; $df$ = 7; $p$ = 0.2905.

# Subgroup Synthesis (Section C) & Correlation Sensitivity Analysis (Section D)

## Assessment of Covariate Influence and Imputation Stability Across Alternative Assumptions

**Table S2 (Section C). Subgroup Analysis Stratified by Pain Measurement Tool (VAS vs. NPRS).**

| Subgroup Stratum / Model Framework | Studies (*k*) | Total *N* | Pooled SMD (*g*) | 95% Confidence Interval | $\tau^2$ | $\tau$ | $I^2$ (%) | Cochran *Q* | *p*-value |
|---|---|---|---|---|---|---|---|---|---|
| **Visual Analogue Scale (VAS)** – Random Effects | 7 | 274 | -0.6610 | [-0.9275; -0.3945] | 0.0150 | 0.1226 | 11.4% | 6.77 (*df* = 6) | 0.3423 |
| **Visual Analogue Scale (VAS)** – Common Effect | 7 | 274 | -0.6676 | [-0.9133; -0.4219] | — | — | 11.4% | 6.77 (*df* = 6) | 0.3423 |
| **Numeric Pain Rating Scale (NPRS)** – Single Trial | 1 | 24 | -0.1058 | [-0.9066; 0.6951] | 0.0000 | 0.0000 | 0.0% | 0.00 (*df* = 0) | — |
| **Subgroup Differences Test (Random Effects)** | **1** | **298** | **Between-group Cochran $Q_b$ = 1.66 (*df* = 1)** | | **—** | **—** | **—** | **1.66** | **0.1973** |
| **Subgroup Differences Test (Common Effect)** | **1** | **298** | **Between-group Cochran $Q_b$ = 1.73 (*df* = 1)** | | **—** | **—** | **—** | **1.73** | **0.1886** |

**Verification of Measurement Instrument Effects & Baseline-to-Endpoint Imputation Stability**

**Section C Methodological Note:** Test for subgroup differences demonstrates no statistically significant disparity between VAS and NPRS ($p = 0.1973$). The lower within-group heterogeneity in the VAS subgroup ($I^2 = 11.4\%$, $\tau^2 = 0.0150$) does not indicate that residual variation is artifactual, nor does it establish comparator-specific superiority.

**Table S3 (Section D). Sensitivity Matrix Across Baseline-to-Endpoint Correlation Coefficients (*r* = 0.30, 0.50, and 0.70).**

| Correlation Parameter Specification | Pooled SMD (Random) | 95% Confidence Interval | *z*-value | *p*-value | $\tau^2$ | $\tau$ | $I^2$ (%) |
|---|---|---|---|---|---|---|---|
| ***r* = 0.30** (Conservative Association) | -0.5842 | [-0.8521; -0.3163] | -4.27 | < 0.0001 | 0.0298 | 0.1726 | 21.2% |
| ***r* = 0.50 (Primary Imputed Benchmark)** | **-0.6046** | **[-0.8707; -0.3384]** | **-4.45** | **< 0.0001** | **0.0259** | **0.1610** | **17.7%** |
| ***r* = 0.70** (Strong Association) | -0.6285 | [-0.8914; -0.3656] | -4.68 | < 0.0001 | 0.0215 | 0.1466 | 14.1% |

**Section D Invariance Verification:** Imputation across correlation values from $r = 0.30$ to $r = 0.70$ produces minimal variance in the pooled point estimate ($\Delta$SMD ≤ 0.04) and fully maintains statistical significance ($p < 0.0001$), confirming inferential robustness.

# Section E: Publication Bias Diagnostics & Complete Leave-One-Out Sensitivity Analysis

### Small-Study Effect Regression, Duval & Tweedie Trim-and-Fill Imputation & Iterative Omission Models

**Table S4 (Section E – Part 1). Small-Study Effects, Egger's Linear Regression Test & Trim-and-Fill Imputation.**

| Diagnostic Assessment / Model Parameter | Value / 95% CI / Dispersion | *p*-value | Analytical Inference |
|---|---|---|---|
| **Egger's Linear Regression Test** | Bias Intercept = 1.6683; $t$ = 1.17, $df$ = 6<br>SE = 1.4293 ($\tau^2_{res}$ = 1.1547) | 0.2874 | Funnel plot symmetry confirmed; no publication bias. |
| **Trim-and-Fill Imputation** (*L*-estimator) | 1 study added; Total $k$ = 9 ($N$ = 312) | — | Filled: Chang et al. mirror (SMD = -1.6604 [-2.717; -0.603]). |
| **Adjusted Random-Effects Synthesis** | **Adjusted SMD = -0.6591 [-0.9539; -0.3644] ($z$ = -4.38)** | **< 0.0001** | **Overall effect remains fully significant and robust.** |

**Trim-and-Fill Variance:** Adjusted $\tau^2$ = 0.0656 [0.0000; 0.8091], $\tau$ = 0.2562, $I^2$ = 33.6% [0.0%; 69.4%], $Q$ = 12.05 ($df$ = 8, $p$ = 0.1489), Adjusted 95% Prediction Interval: [-1.3442; 0.0259].

**Table S5 (Section E – Part 2). Complete Tabular Leave-One-Out Sensitivity Analysis for Pain Intensity.**

| Omitted Study Citation | $k$ | Pooled SMD ($g$) | 95% Confidence Interval | *p*-value | $\tau^2$ | $\tau$ | $I^2$ (%) | Influence & Stability |
|---|---|---|---|---|---|---|---|---|
| Omitting Raeissadat et al., 2018 | 7 | -0.5973 | [-0.8500; -0.3446] | < 0.0001 | 0.0476 | 0.2181 | 27.6% | Stable point estimate (Δ = +0.007). |
| Omitting Porwal et al., 2023 | 7 | -0.5579 | [-0.8063; -0.3094] | < 0.0001 | 0.0059 | 0.0769 | 4.7% | Heterogeneity drops to 4.7%. |
| Omitting Oliveira et al., 2022 | 7 | -0.6676 | [-0.9133; -0.4219] | < 0.0001 | 0.0150 | 0.1226 | 11.4% | Omits single NPRS trial. |
| Omitting Khojasteh et al., 2024 | 7 | -0.6229 | [-0.8708; -0.3749] | < 0.0001 | 0.0496 | 0.2227 | 29.4% | Consistent statistical effect. |
| Omitting Chang et al., 2025 | 7 | -0.6683 | [-0.9092; -0.4274] | < 0.0001 | 0.0000 | 0.0000 | 0.0% | Eliminates all heterogeneity ($I^2$ = 0%). |
| Omitting Ahmed et al., 2016 | 7 | -0.5490 | [-0.8295; -0.2684] | 0.0001 | 0.0414 | 0.2034 | 22.0% | Largest trial (25.0% wt); stays significant. |
| Omitting Choi et al., 2015 | 7 | -0.6029 | [-0.8458; -0.3601] | < 0.0001 | 0.0422 | 0.2053 | 27.1% | Virtually identical pooled point estimate. |
| Omitting Sabir et al., 2016 | 7 | -0.6719 | [-0.9275; -0.4163] | < 0.0001 | 0.0310 | 0.1761 | 19.5% | Effect strengthens slightly. |
| **Complete Dataset (All 8 Trials)** | **8** | **-0.6046** | **[-0.8707; -0.3384]** | **< 0.0001** | **0.0259** | **0.1610** | **17.7%** | **Full Primary Synthesis.** |

**Leave-One-Out Assessment:** Point estimates strictly bounded between -0.5490 and -0.6683, with upper confidence limits consistently below -0.2684. Primary conclusions are entirely independent of any single trial.
